# The Effects of Melatonin on Elevated Liver Enzymes during Statin Treatment

**DOI:** 10.1155/2017/3204504

**Published:** 2017-05-29

**Authors:** Cezary Chojnacki, Aleksandra Błońska, Jan Chojnacki

**Affiliations:** ^1^Department of Clinical Nutrition and Gastroenterological Diagnostics, Medical University, Lodz, Poland; ^2^Department of Gastroenterology, Medical University, Lodz, Poland

## Abstract

Taking statins can cause increase in the level of aspartate and alanine aminotransferase. The aim of this study was to assess the usefulness of melatonin in counteracting the adverse hepatic events from statins.* Methods*. The research program included 60 patients (aged 47–65 years, 41 women and 19 men) with hyperlipidemia taking atorvastatin or rosuvastatin at a dose of 20–40 mg daily. The patients were randomly allocated in two groups. Group I (*n* = 30) was recommended to take the same statin at a standardized daily dose of 20 mg together with melatonin at a dose of 2 × 5 mg. Group II (*n* = 30) patients took statin with placebo at the same dose and time of the day. Follow-up laboratory tests (AST, ALT, GGT, and ALP) were evaluated after 2, 4, and 6 months of treatment.* Results*. In Group I the levels of all enzymes decreased after 6 months, particularly AST, 97,2 ± 19,1 U/L versus 52,8 ± 12,3 U/L (*p* < 0,001); ALT, 87,4 ± 15,6 U/L versus 49,8 ± 14,5 U/L (*p* < 0,001); and GGT, 84,1 ± 14,8 U/L versus 59,6 U/L (*p* < 0,001).* Conclusion*. Melatonin exerts a hepatoprotective effect in patients taking statins.

## 1. Introduction

Statins are widely used in the treatment and prevention of lipid metabolism disorders [1, 2]. They are generally well tolerated but not devoid of side effects [3, 4]. These include, among others, muscular symptoms, arthritis, headaches, and gynecomastia. Myositis and rhabdomyolysis associated with increased activity of creatinine kinase and serum creatinine levels are rare but serious adverse events of statin therapy [5, 6]. The risk of these complications is increased in elderly patients with chronic diseases and in alcohol abusers [7].

Furthermore, statins cause hepatotoxic effect which is observed in several percent of treated patients, usually in the first weeks of the therapy [8–10]. Most frequently it is manifested by asymptomatic increase in the level of aspartate and alanine aminotransferase [11]. This is usually a temporary increase, but in some patients the level of these enzymes exceeds 3 times the normal limit, which is a matter of concern [12, 13]. In such cases, patients expect the decision to discontinue the treatment or to administer hepatoprotective drugs [14]. Acetylcholine, silibinin, phospholipids, and other drugs used for this purpose are not always effective. Therefore, there is still search for alternative drugs for the protection of liver.

In our study melatonin was used for this purpose because previous experimental studies had demonstrated that it protected liver against harmful effects of many toxic agents [15–18] as well as the consequences of ischemia-reperfusion model [19–21].

The liver is an organ in which intensive metabolic and detoxification processes take place. In their course large amounts of reactive oxygen species are generated and they exert a toxic effect on hepatocytes. A complex antioxidant system, in which metabolized there melatonin (pineal and from other sources) is an important part, prevents that [22, 23].

The main melatonin metabolic pathway in the liver is through hydroxylation pathway at the C-6 position by 6-hydroxylase and P450 cytochromes (CYP1A1, CYP1A2, CYP2P19, and CYP1B1 isozymes) [24–26]. The 6-hydroxymelatonin, formed in this process, is conjugated with sulphate and glucuronide to 6-hydroxymelatonin sulphate or glucuronide. In this process melatonin and its metabolites exert high antioxidant activity.

An alternative metabolic pathway includes melatonin oxidation to N-acetyl-formyl-5-methoxykynuramine (AFMK) and N-acetyl-5-methoxykynuramine (AMK). The kynurenine pathway of melatonin metabolism leads to formation of a series of free radical scavengers [27, 28].

Furthermore, melatonin decreases the production of proinflammatory cytokines [29, 30] and inhibits hepatic fibrogenesis [31–34]. Owing to its multidirectional action in liver, apoptosis and necrosis decrease, the integrity is protected, and regeneration is improved [35–38].

The aim of this study was to assess the usefulness of melatonin in counteracting the adverse hepatic events from statins.

## 2. Material and Methods

### 2.1. Patients and Data Collection

The research program included 60 subjects, aged 47–68 years, 41 women and 19 men.  All women were postpostmenopausal. Recruitment and diagnostic tests were conducted in the Department of Gastroenterology, Medical University of Lodz, and Outpatient Consulting Clinic “Gastro” in Lodz.

The research study was performed in the years 2012–2016.

 Inclusion criteria are as follows:

- Hyperlipidemia treated with statins for minimum of 6 months
- At least 2-fold increase in the level of aspartate and alanine aminotransferase found in two consecutive tests
- The persistence of increased aminotransferase levels despite the reduction in the statin dose

At the time of the inclusion of patients in the study, 38 subjects were taking atorvastatin (20 mg) and 26 rosuvastatin at the dose of 40 mg (3 patients), 20 mg (19 patients), and 15 mg (4 patients).

 Exclusion criteria are as follows:

- History of viral hepatitis
- Cholelithiasis
- Body mass index (BMI) > 30 kg/m^2^
- Alcohol abuse
- Familial hypercholesterolemia
- Established hypertension
- Thyroid diseases
- Other organic, metabolic, or mental diseases
- Hormone replacement therapy
- Taking other medications, especially analgesics and psychotropic drugs

### 2.2. Laboratory Tests

The following biochemical parameters using standard automated technique were assessed: blood cells count and levels of bilirubin, aspartate (AST) and alanine (ALT) aminotransferase, gamma-glutamyltransferase (GGT), alkaline phosphatase (ALP), total and LDL and HDL cholesterol, triglycerides, glucose, amylase, lipase, urea, creatinine, acute phase protein, thyroid stimulating hormone (TSH), and follicle-stimulating hormone (FSH) in serum.

### 2.3. Therapeutic Procedures

After inclusion into the study, all patients were recommended the same balanced diet with limited animal fats and simple carbohydrates of caloric content of 1600 kcal. At the same time, they were recommended to continue the treatment with the same statin at a daily dose of 20 mg.

The patients were randomly allocated into two groups. Group I (*n* = 30) was recommended to take statin together with melatonin (LEK-AM, Poland) at a dose of 2 × 5 mg, at 7:00 a.m. and 9:00 p.m. In Group II (*n* = 30) patients took statin with placebo (LEK-AM, Poland) at the same dose and time of the day.

Follow-up laboratory tests (AST, ALT, GGT, ALP, cholesterol, and triglycerides) were evaluated after 2, 4, and 6 months of treatment.

### 2.4. Ethical Procedures

A written consent was obtained from the patients and the Bioethics Committee of the Medical University in Lodz approved the study protocol (RNN/45/12/KB).

Tests were conducted in accordance with the Declaration of Helsinki and with the principles of Good Clinical Practice.

### 2.5. Statistical Analysis

All parameters were checked for normality using the Shapiro-Wilk test. Wilcoxon's rank sum test was used for the comparison of basal treatment differences between each liver enzyme level. Comparison of parameters in four time series was calculated using ANOVA Friedman test. Mann–Whitney *U* test was used for nonparametric data to perform the comparison between groups. Calculations were made using Statistical 9.1 Microsoft Co. software, and statistical significance was established at *p* < 0,05.

## 3. Results

In Group I the initial level of aspartate aminotransferase was 97.2 ± 19.1 U/L. After introduction of melatonin to the treatment this level decreased after 2 months to 77.5 ± 10.9 U/L (*p* < 0.05) and it remained at a similar level after 4 months 66.2 ± 10.3 U/L (*p* < 0.01) and after 6 months 52.8 ± 13.3 U/L (*p* < 0.001).

**Figure 1:**
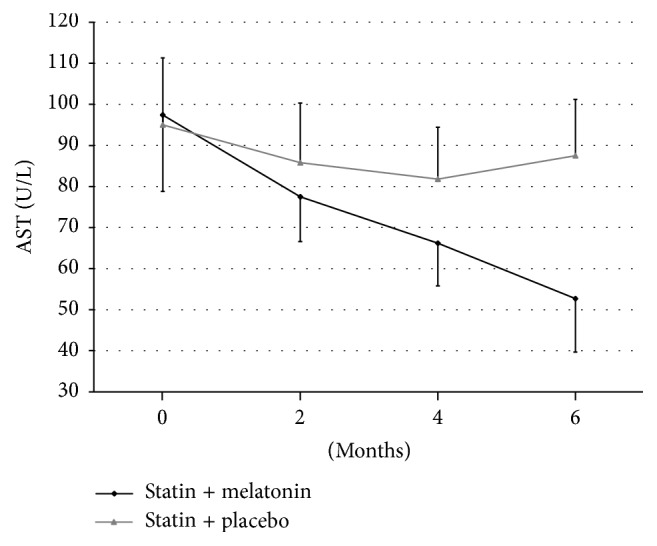
Changes of aspartate aminotransferase (AST) levels during treatment of hyperlipidemia using statin with melatonin or with placebo.

In Group II treated with statin and placebo, the AST level in the same time intervals was, respectively, 95.7 ± 16.3 U/L, 85.8 ± 14.5 U/L, 84.8 ± 12.5 U/L, and 87.5 ± 13.7 U/L; the differences were not statistically significant (Figure 1).

Alanine aminotransferase level was in Group I in the same time intervals, respectively, 87.4 ± 15.6 U/L, 65.2 ± 13.9 U/L (*p* < 0.01), 55.2 ± 11.1 U/L (*p* < 0.001), and 49.8 ± 14.5 U/L (*p* < 0.001).

**Figure 2:**
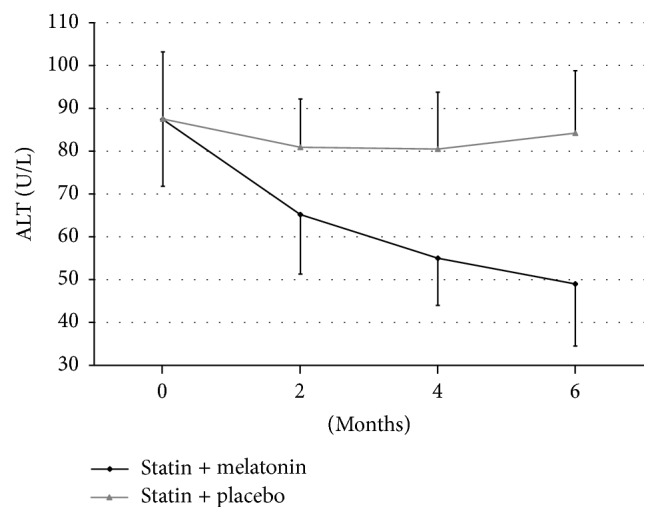
Changes of alanine aminotransferase (ALT) levels during treatment of hyperlipidemia using statin with melatonin or with placebo.

However, in Group II, ALT level did not change significantly during the treatment and it was, respectively, 87.5 ± 15.7 U/L, 80.9 ± 11.3 U/L, 80.5 ± 13.2 U/L, and 84.2 ± 14.6 U/L (Figure 2).

**Figure 3:**
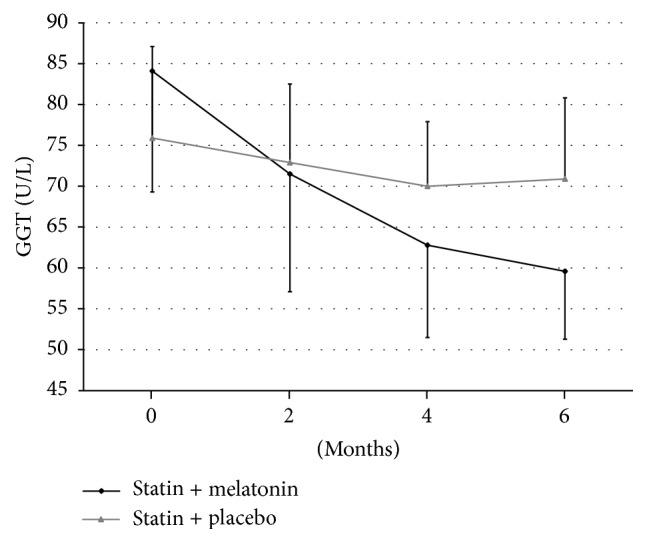
Changes of gamma-glutamyltransferase (GGT) levels during treatment of hyperlipidemia using statin with melatonin or with placebo.

The level of gamma-glutamyltransferase was in group receiving melatonin, respectively, 84.1 ± 14.8 U/L, 71.5 ± 14.3 U/L (*p* > 0.05), 62.8 ± 11.3 U/L (*p* > 0.05), and 59.6 ± 8.3 U/L (*p* < 0.01). In Group II, these values were, respectively, 75.9 ± 11.3 U/L, 72.9 ± 9.7 U/L, 69.9 ± 7.9 U/L, and 70.9 ± 9.2 U/L; the differences were statistically insignificant (Figure 3).

**Figure 4:**
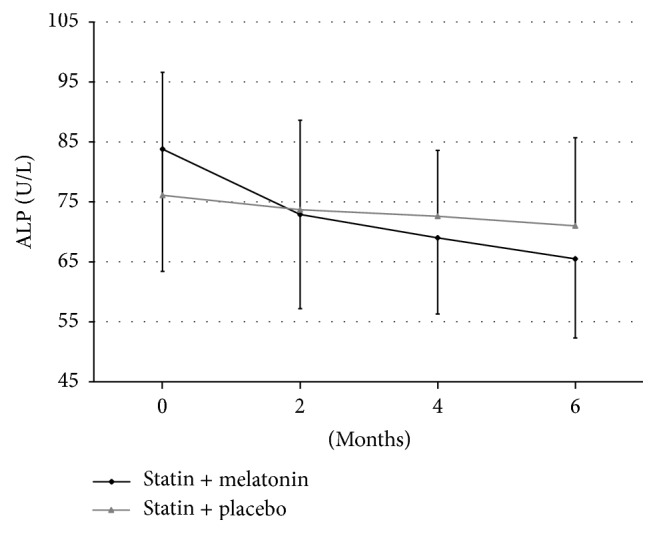
Changes of alkaline phosphatase (ALP) levels during treatment of hyperlipidemia using statin with melatonin or with placebo.

The level of alkaline phosphatase varied in both groups from 58.0 to 142.6 U/L and only in two patients in Group I and two in Group II patients it exceeded the upper limit of normal range of 120 U/L. These values returned to normal after 2 and 4 months of treatment only in Group I (Figure 4).

**Figure 5:**
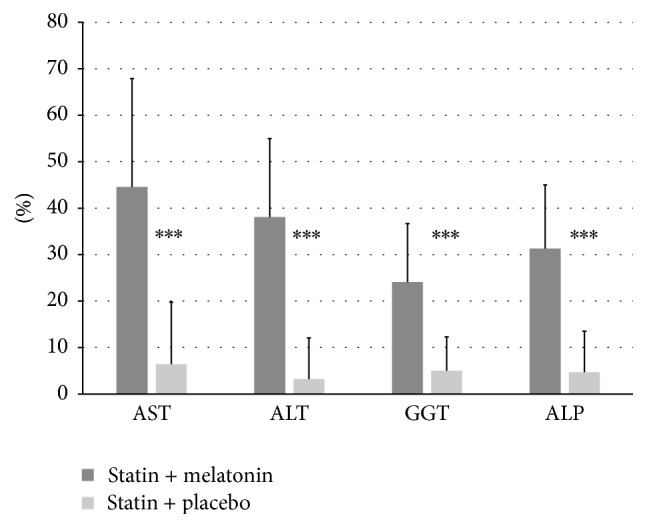
Comparison of the decrease (percentage %) of the aspartate aminotransferase (GGT), alanine aminotransferase (ALT), gamma-glutamyltransferase (GGT), and alkaline phosphatase (ALP) levels before and after 6 months of treatment; all differences statistically significant, ^*∗∗∗*^*p* < 0,001.

After 6 months the decrease of all enzyme levels was significantly higher in Group I compared to Group II (Figure 5).

After 6 months normal AST values were observed in 8 (25,0%), ALT in 9 (28,1%), and GGT in 11 (34,3%) on Group I patients.

In Group II, after 6 months, normal results were found only in 1 patient (3,7%), whereas in 6 of them AST and ALT level increased by the average of 11,3%.

In Group I, the level of total cholesterol decreased after 6 months from 254.2 ± 20.2 mg/dL to 212.6 ± 19.6 mg/dL and in Group II from 245.6 ± 16.4 mg/dL to 226.3 ± 20.7 mg/dL; differences between the groups were statistically significant (*p* < 0.05).

Triglyceride level decreased in Group I from 212,4 ± 20,3 mg/dL to 183,0 ± 14,6 mg/dL and in Group II from 193.6 ± 20,1 mg/dL to 171,4 ± 15,4 mg/dL; the differences between the groups were insignificant (*p* > 0,05).

All drugs were well tolerated. Patients did not complain of any ailments that could result from side effects before treatment or during its continuation. The exception was 6 patients from Group I, who in the first two weeks of taking melatonin felt mild fatigue in the morning hours.

## 4. Discussion

The task of the liver is to transform the lipophilic drugs into hydrophilic compounds, which are excreted in the bile or urine. To this purpose, the drugs undergo oxidation processes by cytochrome P450 isozymes and by conjugation mainly with sulfuric and glucuronic acid or with glutathione. In the oxidation reaction, together with chemically stable metabolites, there may appear reactive metabolites which damage hepatocytes by two main mechanisms.

The first mechanism is immune idiosyncrasy, a reaction involving a metabolite and the enzyme responsible for its formation, usually with an appropriate cytochrome P450. Such a protein complex becomes a new antigen which is transported to the hepatocyte surface. There, cellular and humoral immune response is induced, directed against antigen-presenting cells, which is manifested within a few weeks [39–41].

In the second mechanism, which is a reaction of metabolic hypersensitivity (metabolic idiosyncrasy) immune processes are not present. In this mechanism latency period is usually longer and it may be even several months [42, 43].

The direct drug-induced injury by molecular mechanisms is the opposite of idiosyncrasy. High concentration of a drug or its metabolite leads to the inactivation of many important cytoplasmic or mitochondrial proteins. Drugs can also disturb the processes of mitochondrial fatty acid oxidation or inhibit the activity of the respiratory chain. This results in metabolic changes such as hypertriglyceridemia, decrease in ATP production, and increased production of oxygen free radicals [44–46].

The mechanism of statin-related hepatotoxicity is not clear. It is suggested that statins cause damage to mitochondrial membranes which leads to the leakage of aminotransferases [47, 48]. Statin lipophilicity may play an important role. Statins of low lipophilicity (fluvastatin, rosuvastatin, and atorvastatin) more frequently cause the increase in aminotransferase levels compared to statins of higher lipophilicity (levostatin, simvastatin) [49, 50].

Statins even at low doses may lead to hepatocellular damage; however, this happens more often at high dose [51, 52]. An optimal dose of melatonin, which should be administered in different stages of diseases is a debatable issue. Harpsøe et al. [53] reviewed 392 literature records and found out that the applied melatonin doses ranged from 0.3 mg to 100 mg/daily. In order to control the sleep the most frequently recommended dose was 1–3 mg per night [54, 55]. The dose of 3 and 5 mg was used in the treatment of alimentary tract functional and inflammatory disorders [56–60] and in the treatment of headaches only 10 mg proved to be effective [61].

In the case of patients with nonalcoholic steatohepatitis (NASH), Gonciarz et al. [62, 63] administered melatonin at a dose of 2 × 5 mg for 3 months. The follow-up after 4, 8, and 12 weeks showed the decrease in the level of liver enzymes (AST < ALT and GGT) and this continued for further 12 weeks. Cichoz-Lach et al. [64], also in patients with NASH, used melatonin (2 × 5 mg) for 4 weeks and they also found the decreased level of liver enzymes and triglycerides and proinflammatory cytokines. Cardineli and Hadeland [65] suggested a melatonin dose of 50–100 mg/daily for the regulation of inflammatory and metabolic disorders. Such high single doses of melatonin were given to patients before liver transplantation and its good tolerability and a positive impact on the postoperative condition were demonstrated and it was expressed, among others, by faster drop in AST and ALT compared to placebo [66, 67].

Good tolerability and safety of melatonin result from its pharmacokinetic properties. Andersen et al. [68] administered intravenously 10 or 100 mg of melatonin to 12 healthy volunteers, obtaining its maximum serum concentrations of 185,637 and 1,770,500 pg/mL at T1/2, 43.3 and 46.2 minutes, with the absence of any side effects. The same researchers [69] administered orally 10 mg of melatonin to the volunteers and found its maximum serum concentration of 3550 pg/mL at T1/2, 53.7 min. Similar results of the studies on melatonin pharmacokinetics were obtained by other researchers who used the oral dose of 0.4 mg and 4 mg in older adults [70] and 80 mg in young male volunteers [71]. Thus, a single administration of melatonin raises its level for only a few hours. This justifies the administration of melatonin in divided doses in order to take full advantage of its hepatoprotective effect, particularly in metabolic disorders. Metabolic syndrome belongs to such conditions, since the results of experimental [72–74] and clinical studies [75–80] confirm the beneficial effect of melatonin.

Our results confirm significant hepatoprotective effect of melatonin in patients treated with statins. Medications regulating lipid metabolism are taken chronically and therefore hepatoprotective factors should be used on long-term basis and should be free of side effects; melatonin satisfies such conditions.

## 5. Conclusion

The conclusions from the obtained results support the opinion of many researchers that melatonin can be administered to protect against side effects of other drugs.

## References

[1] Mancini G. B. J., Baker S., Bergeron J. (2011). Diagnosis, prevention, and management of statin adverse effects and intolerance: proceedings of a canadian working group consensus conference. *Canadian Journal of Cardiology*.

[2] Eslami L., Merat S., Malekzadeh R., Nasseri-Moghaddam S., Aramin H. (2013). Statins for non-alcoholic fatty liver disease and non-alcoholic steatohepatitis. *The Cochrane Database of Systematic Reviews*.

[3] Banach M., Rizzo M., Toth P. P. (2015). Statin intolerance – an attempt at a unified definition. Position paper from an international lipid expert panel. *Archives of Medical Science*.

[4] Saxon D. R., Eckel R. H. (2016). Statin intolerance: a literature review and management strategies. *Progress in Cardiovascular Diseases*.

[5] Schalke B. B., Schmidt B., Toyka K., Hartung H.-P., Hayes T. A., Frau L. M. (1992). Pravastatin-associated inflammatory myopathy. *The New England Journal of Medicine*.

[6] Molokhia M., McKeigue P., Curcin V., Majeed A. (2008). Statin induced myopathy and myalgia: time trend analysis and comparison of risk associated with statin class from 1991–2006. *PLoS ONE*.

[7] Bitzur R., Cohen H., Kamari Y., Harats D. (2013). Intolerance to statins: mechanisms and management. *Diabetes Care*.

[8] Thapar M., Russo M., Bonkovsky H. L. (2013). Statins and liver injury. *Journal of Gastroenterology and Hepatology*.

[9] Cadranel J.-F., Seddik M., Loric S., Jeanne S. (2009). Statins: hepatotoxicity and monitoring. *Presse Medicale*.

[10] Jose J. (2016). Statins and its hepatic effects: Newer data, implications, and changing recommendations. *Journal of Pharmacy and Bioallied Sciences*.

[11] Chalasani N., Aljadhey H., Kesterson J., Murray M. D., Hall S. D. (2004). Patients with elevated liver enzymes are not at higher risk for statin hepatotoxicity. *Gastroenterology*.

[12] Armitage J. (2007). The safety of statins in clinical practice. *The Lancet*.

[13] Arca M., Pigna G. (2011). Treating statin-intolerant patients. *Diabetes, Metabolic Syndrome and Obesity*.

[14] Calderon R. M., Cubeddu L. X., Goldberg R. B., Schiff E. R. (2010). Statins in the treatment of dyslipidemia in the presence of elevated liver aminotransferase levels: a therapeutic dilemma. *Mayo Clinic Proceedings*.

[15] Shokrzadeh M., Ahmadi A., Naghshvar F., Chabra A., Jafarinejhad M. (2014). Prophylactic efficacy of melatonin on cyclophosphamide-induced liver toxicity in mice. *BioMed Research International*.

[16] San-Miguel B., Crespo I., Sánchez D. I. (2015). Melatonin inhibits autophagy and endoplasmic reticulum stress in mice with carbon tetrachloride-induced fibrosis. *Journal of Pineal Research*.

[17] Tiao M.-M., Huang L.-T., Chen C.-J. (2014). Melatonin in the regulation of liver steatosis following prenatal glucocorticoid exposure. *BioMed Research International*.

[18] Esteban-Zubero E., Alatorre-Jiménez M. A., López-Pingarrón L. (2016). Melatonin's role in preventing toxin-related and sepsis-mediated hepatic damage: a review. *Pharmacological Research*.

[19] Kang J.-W., Cho H.-I., Lee S.-M. (2014). Melatonin inhibits mTOR-dependent autophagy during liver ischemia/reperfusion. *Cellular Physiology and Biochemistry*.

[20] Gim S.-A., Koh P.-O. (2015). Melatonin attenuates hepatic ischemia through mitogen-activated protein kinase signaling. *Journal of Surgical Research*.

[21] Chen H.-H., Chen Y.-T., Yang C.-C. (2016). Melatonin pretreatment enhances the therapeutic effects of exogenous mitochondria against hepatic ischemia-reperfusion injury in rats through suppression of mitochondrial permeability transition. *Journal of Pineal Research*.

[22] Lane E. A., Moss H. B. (1985). Pharmacokinetics of melatonin in man: first pass hepatic metabolism. *Journal of Clinical Endocrinology & Metabolism*.

[23] Reiter R. J., Rosales-Corral S. A., Manchester L. C., Liu X., Tan D.-X. (2014). Melatonin in the biliary tract and liver: health implications. *Current Pharmaceutical Design*.

[24] Facciolá G., Hidestrand M., Von Bahr C., Tybring G. (2001). Cytochrome P450 isoforms involved in melatonin metabolism in human liver microsomes. *European Journal of Clinical Pharmacology*.

[25] Ma X. C., Idle J. R., Krausz K. W., Gonzalez F. J. (2005). Metabolism of melatonin by human cytochromes P450. *Drug Metabolism and Disposition*.

[26] Letelier M. E., Jara-Sandoval J., Molina-Berríos A., Faúndez M., Aracena-Parks P., Aguilera F. (2010). Melatonin protects the cytochrome P450 system through a novel antioxidant mechanism. *Chemico-Biological Interactions*.

[27] Harthé C., Claudy D., Déchaud H., Vivien-Roels B., Pévet P., Claustrat B. (2003). Radioimmunoassay of N-acetyl-N-formyl-5-methoxykynuramine (AFMK): a melatonin oxidative metabolite. *Life Sciences*.

[28] Galano A., Tan D. X., Reiter R. J. (2013). On the free radical scavenging activities of melatonin's metabolites, AFMK and AMK. *Journal of Pineal Research*.

[29] Akinrinmade F. J., Akinrinde A. S., Amid A. (2016). Changes in serum cytokine levels, hepatic and intestinal morphology in aflatoxin B1-induced injury: modulatory roles of melatonin and flavonoid-rich fractions from Chromolena odorata. *Mycotoxin Research*.

[30] Laliena A., Miguel B. S., Crespo I., Alvarez M., González-Gallego J., Tuñón M. J. (2012). Melatonin attenuates inflammation and promotes regeneration in rabbits with fulminant hepatitis of viral origin. *Journal of Pineal Research*.

[31] Czechowska G., Celinski K., Korolczuk A. (2015). Protective effects of melatonin against thioacetamide-induced liver fibrosis in rats. *Journal of Physiology and Pharmacology*.

[32] Hu W., Ma Z., Jiang S. (2016). Melatonin: the dawning of a treatment for fibrosis?. *Journal of Pineal Research*.

[33] Kang J.-W., Hong J.-M., Lee S.-M. (2016). Melatonin enhances mitophagy and mitochondrial biogenesis in rats with carbon tetrachloride-induced liver fibrosis. *Journal of Pineal Research*.

[34] Shajari S., Laliena A., Heegsma J., Tuñõn M. J., Moshage H., Faber K. N. (2015). Melatonin suppresses activation of hepatic stellate cells through ROR*α*-mediated inhibition of 5-lipoxygenase. *Journal of Pineal Research*.

[35] Cho Y.-A., Noh K., Jue S.-S., Lee S.-Y., Kim E.-C. (2015). Melatonin promotes hepatic differentiation of human dental pulp stem cells: clinical implications for the prevention of liver fibrosis. *Journal of Pineal Research*.

[36] González M. A., del Carmen Contini M., Millen N., Mahieu S. T. (2012). Role of melatonin in the oxidative damage prevention at different times of hepatic regeneration. *Cell Biochemistry and Function*.

[37] Choi H.-S., Kang J.-W., Lee S.-M. (2015). Melatonin attenuates carbon tetrachloride-induced liver fibrosis via inhibition of necroptosis. *Translational Research*.

[38] Stacchiotti A., Favero G., Lavazza A. (2016). Hepatic macrosteatosis is partially converted to microsteatosis by melatonin supplementation in ob/ob mice non-alcoholic fatty liver disease. *PLoS ONE*.

[39] Björnsson E., Jacobsen E. I., Kalaitzakis E. (2012). Hepatotoxicity associated with statins: reports of idiosyncratic liver injury post-marketing. *Journal of Hepatology*.

[40] Hussaini S. H., Farrington E. A. (2007). Idiosyncratic drug-induced liver injury: an overview. *Expert Opinion on Drug Safety*.

[41] Ju C., Reilly T. (2012). Role of immune reactions in drug-induced liver injury (DILI). *Drug Metabolism Reviews*.

[42] Holt M. P., Ju C. (2006). Mechanisms of drug-induced liver injury. *AAPS Journal*.

[43] Tujios S., Fontana R. F. (2011). Mechanisms of drug-induced liver injury: from bedside to bench. *Nature Reviews Gastroenterology & Hepatology*.

[44] Russo M. W., Scobey M., Bonkovsky H. L. (2009). Drug-induced liver injury associated with statins. *Seminars in Liver Disease*.

[45] Stirnimann G., Kessebohm K., Lauterburg B. (2010). Liver injury caused by drugs: an update. *Swiss Medical Weekly*.

[46] Dash A., Figler R. A., Sanyal A. J. (2017). Drug-induced steatohepatitis. *Expert Opinion on Drug Metabolism & Toxicology*.

[47] Dujovne C. A. (2002). Side effects of statins: hepatitis versus 'transaminitis'—Myositis versus 'CPKitis'. *American Journal of Cardiology*.

[48] Tolosa L., Carmona A., Castell J. V., Gómez-Lechón M. J., Donato M. T. (2015). High-content screening of drug-induced mitochondrial impairment in hepatic cells: effects of statins. *Archives of Toxicology*.

[49] Dale K. M., White C. M., Henyan N. N., Kluger J., Coleman C. I. (2007). Impact of statin dosing intensity on transaminase and creatine kinase. *American Journal of Medicine*.

[50] Perdices E. V., Medina-Cáliz I., Hernando S. (2014). Hepatotoxicity associated with statin use: analysis of the cases included in the Spanish Hepatotoxicity Registry. *Revista Espanola De Enfermedades Digestivas*.

[51] Clarke A. T., Johnson P. C. D., Hall G. C., Ford I., Mills P. R. (2016). High dose atorvastatin associated with increased risk of significant hepatotoxicity in comparison to simvastatin in UK GPRD cohort. *PLoS ONE*.

[52] Davidson M. H., Robinson J. G. (2007). Safety of aggressive lipid management. *Journal of the American College of Cardiology*.

[53] Harpsøe N. G., Andersen L. P. H., Gögenur I., Rosenberg J. (2015). Clinical pharmacokinetics of melatonin: a systematic review. *European Journal of Clinical Pharmacology*.

[54] Costello R. B., Lentino C. V., Boyd C. C. (2014). The effectiveness of melatonin for promoting healthy sleep: a rapid evidence assessment of the literature. *Nutrition Journal*.

[55] Huang H., Jiang L., Shen L. (2014). Impact of oral melatonin on critically ill adult patients with ICU sleep deprivation: study protocol for a randomized controlled trial. *Trials*.

[56] Klupińska G., Poplawski T., Drzewoski J. (2007). Therapeutic effect of melatonin in patients with functional dyspepsia. *Journal of Clinical Gastroenterology*.

[57] Mozaffari S., Rahimi R., Abdollahi M. (2010). Implications of melatonin therapy in irritable bowel syndrome: a systematic review. *Current Pharmaceutical Design*.

[58] Chojnacki C., Walecka-Kapica E., Lokieć K. (2013). Influence of melatonin on symptoms of irritable bowel syndrome in postmenopausal women. *Endokrynologia Polska*.

[59] Siah K. T. H., Wong R. K. M., Ho K. Y. (2014). Melatonin for the treatment of irritable bowel syndrome. *World Journal of Gastroenterology*.

[60] Jena G., Trivedi P. P. (2014). A review of the use of melatonin in ulcerative colitis: experimental evidence and new approaches. *Inflammatory Bowel Diseases*.

[61] Gelfand A. A., Goadsby P. J. (2016). The role of melatonin in the treatment of primary headache disorders. *Headache Journals*.

[62] Gonciarz M., Gonciarz Z., Bielanski W. (2010). The pilot study of 3-month course of melatonin treatment of patients with nonalcoholic steatohepatitis: effect on plasma levels of liver enzymes, lipids and melatonin. *Journal of Physiology and Pharmacology*.

[63] Gonciarz M., Gonciarz Z., Bielanski W. (2012). The effects of long-term melatonin treatment on plasma liver enzymes levels and plasma concentrations of lipids and melatonin in patients with nonalcoholic steatohepatitis: a pilot study. *Journal of Physiology and Pharmacology*.

[64] Cichoz-Lach H., Celinski K., Konturek P. C., Konturek S. J., Slomka M. (2010). The effects of l-tryptophan and melatonin on selected biochemical parameters in patients with steatohepatitis. *Journal of Physiology and Pharmacology*.

[65] Cardinali D. P., Hardeland R. (2016). Inflammaging, metabolic syndrome and melatonin: a call for treatment studies. *Neuroendocrinology*.

[66] Schemmer P., Nickkholgh A., Schneider H. (2008). PORTAL: pilot study on the safety and tolerance of preoperative melatonin application in patients undergoing major liver resection: a double-blind randomized placebo-controlled trial. *BMC Surgery*.

[67] Nickkholgh A., Schneider H., Sobirey M. (2011). The use of high-dose melatonin in liver resection is safe: first clinical experience. *Journal of Pineal Research*.

[68] Andersen L. P. H., Werner M. U., Rosenkilde M. M. (2016). Pharmacokinetics of high-dose intravenous melatonin in humans. *The Journal of Clinical Pharmacology*.

[69] Andersen L. P. H., Werner M. U., Rosenkilde M. M. (2016). Pharmacokinetics of oral and intravenous melatonin in healthy volunteers. *BMC Pharmacology and Toxicology*.

[70] Gooneratne N. S., Edwards A. Y. Z., Zhou C., Cuellar N., Grandner M. A., Barrett J. S. (2012). Melatonin pharmacokinetics following two different oral surge-sustained release doses in older adults. *Journal of Pineal Research*.

[71] Waldhauser F., Waldhauser M., Lieberman H. R., Deng M.-H., Lynch H. J., Wurtman R. J. (1984). Bioavailability of oral melatonin in humans. *Neuroendocrinology*.

[72] Korkmaz A., Topal T., Tan D.-X., Reiter R. J. (2009). Role of melatonin in metabolic regulation. *Reviews in Endocrine and Metabolic Disorders*.

[73] Reiter R. J., Tan D.-X., Korkmaz A., Ma S. (2012). Obesity and metabolic syndrome: association with chronodisruption, sleep deprivation, and melatonin suppression. *Annals of Medicine*.

[74] Cardinali D. P., Cano P., Jiménez-Ortega V., Esquifino A. I. (2011). Melatonin and the metabolic syndrome: physiopathologic and therapeutical implications. *Neuroendocrinology*.

[75] Nduhirabandi F., du Toit E. F., Lochner A. (2012). Melatonin and the metabolic syndrome: a tool for effective therapy in obesity-associated abnormalities?. *Acta Physiologica*.

[76] Srinivasan V., Ohta Y., Espino J. (2013). Metabolic syndrome, its pathophysiology and the role of melatonin. *Recent Patents on Endocrine, Metabolic & Immune Drug Discovery*.

[77] Walecka-Kapica E., Klupińska G., Chojnacki J., Tomaszewska-Warda K., Błońska A., Chojnacki C. (2014). The effect of melatonin supplementation on the quality of sleep and weight status in postmenopausal women. *Przeglad Menopauzalny*.

[78] Popov S. S., Shulgin K. K., Popova T. N., Pashkov A. N., Agarkov A. A., de Carvalho M. A. A. P. (2015). Effects of melatonin-aided therapy on the glutathione antioxidant system activity and liver protection. *Journal of Biochemical and Molecular Toxicology*.

[79] Koziróg M., Poliwczak A. R., Duchnowicz P., Koter-Michalak M., Sikora J., Broncel M. (2011). Melatonin treatment improves blood pressure, lipid profile, and parameters of oxidative stress in patients with metabolic syndrome. *Journal of Pineal Research*.

[80] Goyal A., Terry P. D., Superak H. M. (2014). Melatonin supplementation to treat the metabolic syndrome: a randomized controlled trial. *Diabetology and Metabolic Syndrome*.

